# Modeling ripple oscillations in the hippocampus

**DOI:** 10.1186/1471-2202-14-S1-P208

**Published:** 2013-07-08

**Authors:** José R Donoso, Nikolay Chenkov, Richard Kempter

**Affiliations:** 1 Bernstein Center for Computational Neuroscience Berlin, 10115 Berlin, Germany; 2 Institute for Theoretical Biology, Humboldt-Universität zu Berlin, 10115 Berlin, Germany

## 

Sharp wave-ripples (SWRs) are highly synchronous network events displayed by the mammalian hippocampus during slow-wave sleep and immobile resting periods. A SWR event (~100 ms duration) is characterized by fast network oscillations (~200 Hz "ripples") superimposed by a sharp wave, which is a large-amplitude, low-frequency (<10 Hz) signature in the local field potential of the hippocampal CA1 region. Such events are involved in memory consolidation. Understanding the mechanisms that give rise to SWRs can help us to gain insights on the computations being implemented during memory consolidation.

Despite a large amount of electrophysiological data on SWRs *in vivo *(e.g. [1]) and *in vitro *(e.g. [2-4]) and many computational models (e.g. [5] and [6]), the basic mechanisms behind SWR generation remain elusive. Regarding the origin of the high-frequency ripple component during SWRs, two (not mutually exclusive) generative mechanisms have been proposed: First, a rhythmic output of a network of principal cells coupled by gap junctions, presumably between axons of pyramidal cells [5]. Second, an interneuron network coupled by chemical synapses that modulates the firing of pyramidal cells [6]. Modeling studies showed that both mechanisms can give rise to a prominent ripple component in the 200 Hz range. These two mechanisms could not only coexist but also be differentially expressed during SWR generation.

Here we explore the oscillatory behavior of an *in-silico *model of a CA1 interneuron network to mimic *in-vitro *recordings from CA1 slices in which excitatory synaptic transmission is blocked and SWRs are induced by application of brief potassium puffs, which transiently excite neurons [3]. In such a preparation, the two proposed ripple generating mechanisms are decoupled and can be studied in isolation. Focusing on a model of a random interneuron network, we found that the power of ripple oscillations is stable for a wide range of the strength of the recurrent inhibitory coupling. A reduction of inhibitory conductance leads to a slight increase in network frequency, which is in line with [4]. Furthermore, larger reductions of inhibitory coupling leads to an abrupt decay of ripple power (see also [3]). Thus, some of the phenomenology of SWRs *in vitro *can be described in terms of chemical synaptic inhibition.

## References

[B1] Ylinen A Bragin A Nadasdy Z Szabo I Sik A Buzsáki G Sharp wave-associated high-frequency oscillation (200 Hz) in the intact hippocampus: network and intracellular mechanisms J Neurosci 1995 15 30 46 7823136 10.1523/JNEUROSCI.15-01-00030.1995 PMC6578299

[B2] Csicsvari J Hirase H Czurkó A Mamiya A Buzsáki G Fast network oscillations in the hippocampal CA1 region of the behaving rat J Neurosci 1999 19 RC20 10436076 10.1523/JNEUROSCI.19-16-j0001.1999 PMC6782850

[B3] Maier N Tejero-Cantero A Dorrn A Winterer J Beed P Morris G Kempter R Poulet J Leibold C Schmitz D Coherent phasic excitation during hippocampal ripples Neuron 2011 72 137 152 10.1016/j.neuron.2011.08.016 21982375

[B4] Nimmrich V Maier N Schmitz D Draguhn A Induced sharp wave-ripple complexes in the absence of synaptic inhibition in mouse hippocampal slices J Physiol 2005 563 3 663 670 10.1113/jphysiol.2004.079558 15661820 PMC1665611

[B5] Behrens CJ van den Boom LP Heinemann U Effects of the GABA_A _receptor antagonists bicuculline and gabazine on stimulus-induced sharp-wave ripple complexes in adult rat hippocampus in vitro Eur J Neurosci 2007 25 2170 2181 10.1111/j.1460-9568.2007.05462.x 17419756

[B6] Traub RD Schmitz D Jeffreys JG Draguhn A High-frequency population oscillations are predicted to occur in hippocampal pyramidal neuronal networks interconnected by axo-axonal gap junctions Neuroscience 1999 92 407 426 10.1016/S0306-4522(98)00755-6 10408594

[B7] Brunel N Wang XJ What determines the frequency of fast network oscillations with irregular neural discharges? I. Synaptic dynamics and excitation-inhibition balance J Neurophysiol 2003 90 415 430 10.1152/jn.01095.2002 12611969

